# The Nakuru eye disease cohort study: methodology & rationale

**DOI:** 10.1186/1471-2415-14-60

**Published:** 2014-05-01

**Authors:** Andrew Bastawrous, Wanjiku Mathenge, Tunde Peto, Helen A Weiss, Hillary Rono, Allen Foster, Matthew Burton, Hannah Kuper

**Affiliations:** 1 International Centre for Eye Health, Department of Clinical Research, Faculty of Infectious and Tropical Diseases, London School of Hygiene and Tropical Medicine (LSHTM), Keppel Street, London WC1E 7HT, UK; 2 NIHR Biomedical Research Centre, Moorfields Eye Hospital NHS Foundation Trust and UCL Institute of Ophthalmology, 162 City Road, London EV1V 2PD, UK; 3 Rwanda International Institute of Ophthalmology, P.O. Box 312, Kigali, Rwanda; 4 The Fred Hollows Foundation, P.O. Box 8683, 00200 Nairobi, Kenya; 5 MRC Tropical Epidemiology Group, Faculty of Epidemiology & Population Health, London School of Hygiene and Tropical Medicine (LSHTM), Keppel Street, London WC1E 7HT, UK; 6 Kitale and Zonal eye surgeon, North Rift, Kenya; 7 International Centre for Evidence in Disability, London School of Hygiene and Tropical Medicine (LSHTM), Keppel Street, London WC1E 7HT, UK

**Keywords:** Cohort study, Longitudinal, Eye disease, Africa, Kenya, Cataract, Glaucoma, Age related macular degeneration, Diabetic retinopathy, Refractive error, Incidence, Progression

## Abstract

**Background:**

No longitudinal data from population-based studies of eye disease in sub-Saharan-Africa are available. A population-based survey was undertaken in 2007/08 to estimate the prevalence and determinants of blindness and low vision in Nakuru district, Kenya. This survey formed the baseline to a six-year prospective cohort study to estimate the incidence and progression of eye disease in this population.

**Methods/Design:**

A nationally representative sample of persons aged 50 years and above were selected between January 2007 and November 2008 through probability proportionate to size sampling of clusters, with sampling of individuals within clusters through compact segment sampling. Selected participants underwent detailed ophthalmic examinations which included: visual acuity, autorefraction, visual fields, slit lamp assessment of the anterior and posterior segments, lens grading and fundus photography. In addition, anthropometric measures were taken and risk factors were assessed through structured interviews. Six years later (2013/2014) all subjects were invited for follow-up assessment, repeating the baseline examination methodology.

**Discussion:**

The methodology will provide estimates of the progression of eye diseases and incidence of blindness, visual impairment, and eye diseases in an adult Kenyan population.

## Background

The most recent global estimates suggest 285 million people worldwide are visually impaired, of whom, 39 million are blind [1]. The WHO defined Africa region has 26 million people with visual impairment (VI) of whom 6 million are blind. The continent also has the greatest disparity between numbers blind and number of ophthalmologists per million people [2], and therefore the greatest need for scaling up services.

In recent years several cross-sectional surveys have been undertaken across Africa to estimate prevalence and causes of blindness [3-16]. Whilst this information has been vital in planning services where resources and provision of healthcare are limited, data on incidence and rates of progression of eye disease are needed to allow long-term planning. To date, no longitudinal, population-based studies of eye disease have been undertaken in Africa, and there have been only ten worldwide, predominantly in high-income settings (Table 1) [17-26].

**Table 1:** Population-based cohort studies of eye disease (not exhaustive)

**Study**	**Location**	**Year commenced**	**Years of follow up**	**No of participants**	Reference*
Beaver Dam Eye Study	USA	1988	Baseline	4926	[17]
			5	3684	
			10	2764	
			15	2119	
Blue Mountain Eye Study	Australia	1992	Baseline	3654	[18]
			5	2335	
			10	1952	
Rotterdam Study	Netherlands	1990	Baseline	6418	[19]
			2	4953	
			6.5	3406	
			11	2387	
Copenhagen City Eye Study	Denmark	1986	Baseline	946	[20]
			14	359	
Barbados Eye Study	Barbados	1987	Baseline	4631	[21]
			4	3427	
			9	2793	
Pathologies Oculaires Liees a L’Age	France	1995	Baseline	2584	[22]
			3	1642	
Melbourne Visual Impairment Project	Australia	1992	Baseline	5147	[23]
			5	3271	
Hisayama Study	Japan	1998	Baseline	1482	[24]
			5	961	
			9	(1401>40 yrs)	
Reykjavik Eye Study	Iceland	1996	Baseline	1045	[25]
			5	846	
Los Angeles Latino Eye Study	USA	2000	Baseline	6357	[26]
			4	4658	

*Only one reference per study given.

The current study was undertaken in Nakuru district (now Nakuru County), which is the main district of Kenya’s largest province, the Rift Valley and has a population of 1.6 million. Nakuru district is broadly representative of Kenya in terms of ethnic diversity and economic activities. In 2004, a Rapid Assessment of Avoidable Blindness (RAAB) was completed in Nakuru district, to estimate the prevalence and causes of avoidable blindness and VI in the population of those 50 years and over [7]. A subsequent more comprehensive study was planned in the same region as a consequence of this survey to estimate causes and risk factors for those with visual impairment as well as those with non-visually impairing eye disease, with a particular focus on posterior segment eye disease [6].

Fieldwork was carried out in 2007 and 2008, during the course of which 4414 participants (a response rate of 88.1%) aged 50 years and above underwent ophthalmic and/or general examinations.

The prevalence of blindness and visual impairment [6], glaucoma, age-related macular degeneration (AMD) [27], diabetic retinopathy (DR), cataract, refractive error (RE) [28,29] and cardiovascular diseases [30,31] were assessed. This 2007/08 survey forms the baseline to cohort described here.

The overall aim of this cohort is to estimate the incidence, progression and risk factors for the development of blindness/visual impairment and their leading causes in a Kenyan adult population.

### Objectives

#### Incidence

To estimate the age- and sex- specific incidence of visual impairment (VA < 6/12) and blindness (VA < 3/60) (all causes) in a Kenyan adult population.

To estimate the age- and sex- specific incidence of cataract, RE, glaucoma, AMD and DR.

#### Causes & risk factors

To identify the causes and risk factors for incident visual impairment and blindness from specific diseases investigated (specifically focusing on demographic, anthropometric, behavioural, and vascular risk factors).

#### Progression

To estimate the risk of progression of Cataract, RE, Glaucoma, AMD and DR among cases detected at baseline.

#### Treatment & progression outcome

To describe the outcome of treatment for cataract, RE, glaucoma or DR among cases detected at baseline.

To describe the progression of untreated eye disease among cases detected at baseline.

## Methods/Design

This paper describes the definitions, eligibility criteria, follow-up procedures, visual acuity (VA) measurements, anthropometry and clinical examination procedures adopted for the study.

### Baseline study population - sample size

The sample size of 5000 participants required for the baseline survey was calculated based on an expected prevalence of VA < 6/12 in the better eye due to posterior segment eye diseases (PSED) of 3.0% among those aged ≥50 years, a required precision of 0.5% (i.e. a 95% confidence interval [CI] of 2.5%-3.5%), a design effect of 1.5, and a response rate of 90%. (Epi Info 6.04, Centers for Disease Control and Prevention, Atlanta, GA). We selected 100 clusters each of 50 participants.

### Sampling strategy and recruitment

**Figure 1:**
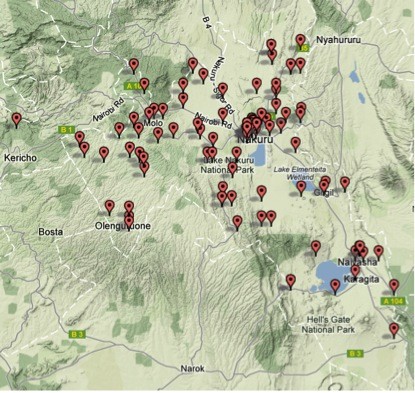
Map showing the 100 study locations in the Nakuru County, Kenya.

Recent census data for Kenya were not available [32], and therefore electoral role lists that were renewed in 2006 in preparation for the 2007 general elections were used as the sampling frame for this baseline survey. The population size was updated for the year 2007 using a population growth rate of 2.7% per year [33]. One hundred clusters were selected with a probability proportional to the size of the population (Figure 1). A cluster was defined as the area served by the polling station.

Households were selected within clusters using a modified compact segment sampling method [34]. Each cluster was divided into segments so that each segment included approximately 50 people aged ≥ 50 years. For instance, if a cluster included 200 people aged ≥ 50 years then it was divided into four segments. One of the segments was chosen at random by drawing lots and all households in the segment were sequentially sampled, until 50 people aged ≥ 50 years were identified. An eligible individual was defined as someone aged ≥ 50 years living in the household for at least three months in the previous year. Age was determined using the subject’s testimony, national identity cards and a calendar of historic events. If the segment did not include 50 people aged ≥ 50 years then another segment was chosen at random and sampling continued until 50 were reached. If after enumerating individual number 49 the next household had more than one person aged ≥50 all were enumerated and invited for examination.

### Baseline findings

In total, 4381 participants underwent complete (ophthalmic and general) examination at baseline across 100 clusters. The prevalence of blindness was 1.6% (95% CI: 1.2-2.1%) [6].

### Follow-up

A pilot follow up retraced 438 participants from 10 of the 100 clusters in 2008, a mean of 1.5 years from baseline, and 408 (79%) were successfully retraced to give an estimated 4.2% loss per year.

### Retracing at follow-up - advance team

Approximately one week before the follow-up examination clinic was planned for a given cluster, a field officer studied the maps of the village and made phone contact with the village chief or guide to arrange the visit. At the planning visit a list of study participants were given to the chief and a local village guide was recruited to assist location of the study participants. At this visit the examination site was established and identification of amenities such as electricity, water and road access were made. Two days prior to the clinic, the field officer reminded chiefs of the visit by phone and notified them and the guide of the advance team’s arrival.

On the day prior to the examination clinic, the Advance Team visited homes of baseline participants and confirmed their identity using National Identity cards. The two advance teams comprised of one nurse, one field officer and a driver or public transport. During this visit they performed the following duties:

○ Located individuals with assistance from the guide, phone numbers when available and previously recorded GPS locations using a Garmin Oregon 450 Satellite Navigation device.

○ Explained details of the exam and obtain written/thumb print informed consent for examination

○ Informed selected participants about location and time for examination

### Registration

On the examination day, the advance team confirmed the identity of participants against their records from the previous day and against data from baseline (age, date of birth, name, and identity cards). Each participant was given a questionnaire, which was completed by the examiners as they move from station to station.

### Examination procedures

Examinations were performed as per baseline unless otherwise indicated in Table 2.

**Table 2:** Instruments used at baseline and follow-up for examination, including rationale for change where appropriate

**Procedure**	**Baseline Instrument (2007/08)**	**Follow-up instrument (2013/14)**	**Rationale for change**
Near Vision Test	Continuous Text “Read in Style®” diopter chart	Unchanged	N/A
Personal Interview	Questionnaire developed by the survey ophthalmologist (WM)	Questionnaire developed by the survey ophthalmologist (AB) see Additional file 1: Appendix	N/A
Weight	The Seca 761 Medical Class 4 Scales mechanical ground scale (Williams Medical Supplies, London)	Tanita Segmental Body Composition Monitor	Combined weight and bioimpedence device – approved for medical studies
Bioimpedence	Not performed	Tanita Segmental Body Composition Monitor	Combined weight and bioimpedence device – approved for medical studies
Height	Leicester Height Measure (Stadiometer) (Chasmors Ltd, London)	SECA Height Measure	Better stability on uneven grounds
Waist and Hip circumference	Chasmors WM02 Body Tape measure	SECA Measuring tape	Availability
Blood pressure	Omron® Digital Automatic Blood Pressure Monitor Model HEM907	Unchanged	N/A
Visual Acuity	ETDRS LogMAR chart	Unchanged	N/A
Auto refraction	Topcon® Auto refractor RM8800	Welch Allyn SureSight	Improved portability
Corrected Visual Acuity	Frames and standard refraction lenses	Unchanged	N/A
Undilated eye exam including imaging (SL-OCT)	Haag-Streit® Slit lamp BD900 with SL-OCT	Haag-Streit® Slit lamp BM900 – no SL-OCT	Availability
Tonometry	Haag Streit® Goldmann Applanation tonometer on above slit lamp	Haag Streit® Goldmann Applanation tonometer on above slit lamp	N/A
Gonioscopy	Not performed	Four-mirror non-coupling gonioscopy lens (Zabby’s)	For glaucoma sub-typing and angle evaluation in normal population
Visual fields	Humphrey Field Analyzer II -720 *i* series(Zeiss®)	Henson 8000 Visual Field Analyser (TopCon, Inc)	Deemed more suitable for epidemiological data collection
Pupil Dilation	Mydriacyl drops (Alcon®)	G. Tropicamide 1% + G. Phenylepherine 2.5% (Minims)	Single units and better shelf life
Blood sugar	Accutrend GCT and test strips (Roche®)	OneTouch Select, Lifescan	Availability. Approved for medical studies
Blood Cholesterol	Accutrend GCT and test strips (Roche®)	Not performed	Cost prohibited inclusion
HbA1c	Not performed	(A1C Now+, Bayer)	Increase accuracy of Diabetes Mellitus diagnosis as participants non-fasted
Examination of anterior and posterior segments through a dilated pupil	90D lens and slit lamp(Volk®)	Superfield and 60D Lens (Volk) and Slit Lamp	Study ophthalmologist preference
Retinal Photo	Topcon® NW6S Non Mydriatic camera model	Haag-Streit DRS Retinal Camera	Suitability for travel and ease of use.

Details of each examination station are provided below including differences, if there were any, between baseline and follow-up.

### Anthropometry

A nurse performed and recorded measures of participants: height; weight; waist and hip circumference, and three measures of blood pressure, each 5 minutes apart. In addition, at follow-up, bioimpedence (Tanita Segmental Body Composition Monitor) was performed.

At baseline, capillary blood was taken from all participants for random blood glucose and cholesterol. At follow-up, no blood for cholesterol was collected and in addition, subjects with a random blood sugar greater than 11.1 mmol/L (IDF guidance at time of baseline study), those with known diabetes (regardless of random measure), evidence of diabetic retinopathy on retinal imaging and a subset (chosen randomly within each cluster) with random glucose between 7-11 mmol/L had an additional capillary blood HbA1c (A1C Now+, Bayer).

### Interview

An interviewer performed the structured interview in the participant’s preferred language covering i) demographic details including; name, year of birth, ethnicity and education level; ii) past medical and ocular history including medical or ophthalmic medication or surgery and relevant family history; iii) relevant risk factors including; smoking and tobacco consumption and alcohol intake; iv) socioeconomic status based on job, housing conditions, ownership of material goods and livestock which is translated in to a score based on previous work in the same population [35]. (See Additional file 1: Appendix for Questionnaire/Data Entry Booklet).

### Visual acuity

A clinical officer determined whether the study participant:

a) Attends wearing distance correction glasses

b) Owns distance correction glasses but failed to bring them

c) Does not have any distance glasses

d) Routinely uses reading glasses

e) Attends wearing aphakic glasses

Visual acuity (VA) was measured using a back-illuminated modified LogMAR reduced tumbling E chart [36,37], which has been used in previous population based studies [38,39].

### Autorefraction

All subjects, regardless of VA underwent autorefraction using the Topcon® Auto refractor RM8800 at baseline and the hand held SureSight autorefractor (Welch Allyn) at follow-up, following manufacturers guidelines. Any subject recording an acuity of ≤24 optotypes, <6/9 equivalent (with or without glasses) underwent best corrected visual acuity. The refraction measure recorded for each eye was mounted in the trial frames using trial lenses (rounded up or down to the nearest 0.25 diopters). Visual acuity was then re-measured to give an estimate of the “corrected visual acuity” in each eye individually. When autorefraction results were not available, the pinhole method was used to estimate corrected visual acuity. A subset of participants also underwent manual refraction by a visiting optometrist for five clusters to validate the accuracy of the autorefractor.

### Visual field assessment

At baseline, all individuals with suspect or abnormal discs on clinical examination underwent automated visual field testing. The Humphrey® Field Analyzer II - 720*i* series (Carl Zeiss Ophthalmic Systems, Inc.) was used. A suspect or abnormal disc was defined as a vertical cup/disc ratio (VCDR) of 0.7 or above; optic disc cupping asymmetry between the eyes of more than 0.2 VCDR; or any other abnormal features. A random sample of five individuals per cluster (10%) also underwent visual field testing to provide normative data.

Participants performed the Swedish Interactive Thresholding Algorithm (SITA) STANDARD 24–2. SITA Fast was used to determine the threshold level in all participants having visual field analysis. Appropriate corrective lenses for refractive errors were used when needed. An automated fixation monitor was used throughout. If the SITA fast test was reliable (following manufacturers guidelines) the SITA standard test was performed. If the SITA fast was unreliable then this was repeated once. If it remained unreliable then no further testing was done.

At follow-up, a different strategy for visual field testing was used: All subjects with VA equivalent to > =6/60 Snellen underwent automated visual field testing by a trained visual field technician using the Henson 8000 Visual Field Analyser (TopCon, Inc.) The multiple stimulus suprathreshold test was used following manufacturers guidelines (Screening test - 26 test locations). When one or more spots were missed, the 26-point test was repeated for that eye. If any missed spots re-occurred on the second time of testing the test for that eye was extended to 68 test locations. This machine and strategy were used in preference to the baseline methods due to feedback from both patient’s and tester at baseline. Patient’s found the baseline testing protocol difficult to understand and the time required to complete the test meant only a sub-sample of the population could be investigated.

### Slit lamp biomicroscopy examination

Undilated (anterior segment) and dilated (posterior segment) slit lamp biomicroscopy examination were performed on all participants by the study ophthalmologists (WM at baseline, AB at follow-up) using a Haag-Streit BD 900 Slit Lamp (BM 900 at follow-up) and Volk condensing lenses (90D at baseline, Superfield and 66D at follow-up).

#### Anterior segment

The anterior segment of the eye was assessed for the presence of signs of trachoma. In addition at follow up examination included grading of corneal scarring, pterygium, secondary glaucoma, evidence of past or active uveitis, or evidence of surgery. The angle at baseline and follow-up was assessed using the Van Herick Test [40] and direct visualization of the angle using gonioscopy (performed after intraocular pressure, see below) was performed at follow-up.

At follow-up, the ophthalmologist using a bright LED pen torch tested for the Relative Afferent Pupil Defect (RAPD). RAPD was recorded as present or absent. If present it was sub-categorised in to “subtle” or “definite”.

#### Intraocular pressure

Goldmann Applanation Tonometry (GAT) was used to measure intraocular pressure (IOP). A drop of Proxymethacaine and fluorescein (minims) were instilled to each eye. After 20 seconds the GAT was used in combination with a slit lamp to measure the IOP in each eye. The GAT’s calibration was checked as per manufacturers instructions on a daily basis by the study ophthalmologist, if found to be inaccurate, the spare GAT was used whilst the original was returned to the factory for calibration. One reading was taken from each eye and the GAT was disinfected between patients.

#### Gonioscopy

Assessment of the opening angle of participants’ right and left eyes was made using a four-mirror gonioscopy lens (Zabbys). This lens does not require coupling fluid and was chosen to minimize impact on the quality of retinal photographs. Angles were recorded using standard Shaffer grading [41] and further classified as “open”, “occludable” or “closed” based on standard referral criteria. Occludable angles are defined as: pigmented trabecular meshwork not visible in 3⁄4 or more of angle circumference in primary position without manipulation, in presence of low illumination. If the patient could not cooperate with gonioscopy, the Van Herick (VH) technique [40] was used for grading.

### Dilated slit lamp examination

Pharmacologic dilation of the subject’s pupils was achieved by using tropicamide 1% (Mydriacyl) with phenylephrine hydrochloride 2.5% if needed. Dilation was not performed in subjects deemed at risk of narrow angle closure (inability to visualise at least 180**°** of posterior pigmented trabecular meshwork on non-indentation gonioscopy [42]). At risk subjects were referred to the Nakuru Eye Unit for prophylactic laser peripheral iridotomies.

#### Lens

The WHO simplified system for lens grading was used [43] following standard protocols. The lens was also examined for position, the presence of hyper mature (Morgagnian) cataract, and previous lens surgery (aphakic or pseudophakic). A red reflex lens image was taken when each participant was having retinal photographs. At follow-up, pseudophakic participants were assessed for the presence or absence of posterior capsular opacification and, if present, whether it entered the visual axis.

#### Optic disc

The optic nerve head was examined using a 90 Diopter Lens (Volk) at the slit lamp at baseline and a 66 Diopter lens (Volk) at follow-up. The clarity of the optic nerve head was determined and graded as clear, hazy or no view. Among subjects in whom an adequate view of the disc was obtained, the VCDR was estimated and recorded for each eye. Other glaucomatous changes were recorded and non-glaucomatous characteristics such as optic atrophy and optic pits were also recorded.

#### Macula

The macula was examined using a 90 Diopter Lens (Volk) at the slit lamp at baseline and a 66 Diopter or Superfield lens (Volk) at follow-up. The view of the macula was recorded as clear, hazy or no view. DR was clinically graded and recorded as absent, non proliferative, proliferative and end stage or maculopathy (macula oedema) [44]. The presence of drusen, hypo or hyper pigmentation, dry or geographic atrophy and neovascular changes were also recorded.

### Fundus photography

An Ophthalmic Assistant performed digital photography of the lens and fundus on all study participants using a Topcon® NW6S Non Mydriatic camera model at baseline and DRS Digital Fundus Camera (Haag-Streit) at follow-up. The study ophthalmologist checked images were of sufficient quality for grading in the absence of prominent media opacities. An anterior segment co-axial photograph was taken for lens grading from each eye. Two 45**°** fundus photographs were taken in each eye, one optic disc centered and the other macula centered. Images were then securely uploaded to the Moorfields Reading Centre for review and grading for image quality, the presence or absence of pathology and the severity of pathology when present.

Note: The gold-standard for grading of DR, AMD and optic disc changes is based on retinal photographs and not clinical assessment. Clinical examination was performed as a backup to equipment failure and a comparison of clinical and image based grading can be compared in the analysis stage and factored in the scenario by which a number of participants only have clinical grading available.

### Data management and analysis

A patient record was completed for each participant and crosschecked for errors by the project field coordinator. Patient records were scanned to create a digital backup and then data were entered into an EpiData database (with built in range and consistency checks) independently by two data clerks and validated by the study ophthalmologist to reconcile any differences. Further data cleaning and all statistical analyses were conducted using STATA 10.0 (StataCorp LP, Texas, USA).

The visual fields PDF print outs and raw data were sent securely to Moorfields Eye Hospital for grading along with the fundus and anterior segment images at baseline and follow-up. All image and visual field data were backed up on local devices and external hard drives. All images were first examined for quality and categorized as excellent, good, fair, borderline and ungradeable. If the images were ungradeable the clinical diagnosis was used. For gradable images the retina and optic disc were reviewed, and a diagnosis made based on the appearance of the image e.g. diabetic retinopathy, toxoplasmosis, onchocerciasis, age related macular degeneration, myopic fundus, glaucoma, optic atrophy or other retinal pathology. VCDR was measured and all images were graded for the absence/presence and stage of DR and ARMD. The graders graded the images for as many disease categories as possible, and so if it was feasible to grade an image for optic disc abnormality but not for ARMD, then the grader completed the optic disc grading only. A senior grader verified a random 10% of images that were graded as normal as well as all abnormal images to ensure quality assurance. The graders re-graded a random selection of images with a minimum of 14-days interval to allow for intragrader reliability to be established.

Definitions used for analysis are detailed in Table 3.

**Table 3:** Definitions of disease incidence and progression

**Disease**	**Incidence**		**Progression**	
**Definition**	**At risk**	**Cases**	**At risk**	**Cases**
**Blindness and Visual Impairment (VI)**	Blind: Persons with VA of ≥3/60 in the better eye at baseline.	Persons who have VA of <3/60 in the better eye at follow up who had ≥3/60 in the better eye at baseline	Categorical changes in visual acuity between: Normal; Mild VI; Moderate VI; Severe VI; Blind, with a minimum of two line Snellen equivalent change in VA.	
**Cataract**	Persons without evidence of cataract at baseline based on WHO simplified cataract grading systems	Persons with evidence of cataract at follow-up based on WHO simplified cataract grading systems [45] who did not have evidence at baseline	Persons with evidence of any grade of cataract at baseline based on WHO simplified cataract grading systems	Persons who increase by two or more severity grades in each sub-type of cataract.
**Primary open angle glaucoma**	Persons without glaucoma in either eye at baseline based on ISGEO [46] criteria	Persons who develop ISGEO classification 1, 2 or 3 glaucoma by the 6-year follow-up point	Glaucoma or glaucoma suspect case at baseline	Definite, disc or field progression. See below* [47]
**Age-related macula degeneration**	Persons who did not have any evidence of AMD at baseline in both eyes	Persons with evidence of early, late or specific AMD lesions	AREDS [48] step 9 or less (no AMD or early AMD) at baseline.	2-or-more-step increase in combined AREDS score from baseline in persons with gradable fundus photographs at both time points.
**Diabetic retinopathy**	Persons with diabetes and free of retinopathy at baseline and persons developing DM by follow up.	Persons with signs of DR (ETDRS) [49]	Persons with diabetes and minimal or moderate DR at baseline	(1) Persons who develop severe DR by the 6-year follow-up
		CSME and incidence of proliferative or severe DR^**ϒ**^		
				(2) Increase by ≥ 3 steps on the ETDRS Severity Scale or development of proliferative DR necessitating photocoagulation therapy or vitrectomy

*Definite progression will be defined as those with a combination of ≥0.2 VCDR increase in either eye and/or ≥0.2 VCDR asymmetry between the two eyes with a corresponding progression on the visual field test defined as (TBC – either “expert analysis” or an arbitrary objective measure).

Note: More specific definitions will be provided in subsequent papers.

### Quality assurance procedures

#### Training

Inter observer variations (IOV) assessments were performed in the training phase. IOV assessments on anthropometric variables were done by having the two nurses perform repeat measuring of 50 subjects. IOV of visual acuity were undertaken by having the ophthalmic clinical officer (OCO) and ophthalmic nurse repeat measures of 50 subjects (half normal vision and half visual impairment). IOV of undilated examinations were done by repeat measure of 50 subjects (half normal vision and half visual impairment) by a visiting ophthalmologist and study ophthalmologist at the beginning of the baseline survey and again in the middle. IOV of dilated exams were done by repeat measure of 50 subjects (half normal vision and half visual impairment) by a visiting ophthalmologist and study ophthalmologist at baseline. Retraining was done where IOV scores indicated poor comparability (kappa < 0.5).

At follow-up, four weeks of training in November/December 2012 was completed for the study team members on all equipment and study protocols. Three pilot clusters examining over one hundred people were completed prior to commencing the study.

Standard Operating Procedures (SOP) detailing follow-up survey methodology for each examination station were prepared and read by all study team members. The SOP was used in training and for reference during fieldwork. Supervisory visits were made to the field site (HK and MJB) to monitor practices and ensure standard protocols were being followed.

### Non-responders at follow-up

Participants who were examined at baseline and eligible for follow-up assessment but who did not attend the examination clinic were contacted to determine the reason for their absence. Participants who were not locatable for the examination were categorized as non-responders and their reason for absence determined through available phone contact, neighbors and village guides as, “deceased”, “moved away”, or “unknown”.

### Service provision

All participants identified with treatable disease in the study were offered appropriate care including free surgery and transport to the Rift Valley General Provinical Hospital or St Mary’s mission hospital, Elementita. Specific cases requiring other services were referred to the Kikuyu Eye Unit. A trained ophthalmic nurse or Ophthalmic Clinical Officer (OCO) discussed the diagnosis and the treatment options available to subjects diagnosed with untreatable eye disease. As well as study participants, non-study attendees were examined and treated by the study team.

### Ethical approval

The study adhered to the tenets of the Declaration of Helsinki and was approved by the Ethics Committee of London School of Hygiene and Tropical Medicine at both baseline and follow-up. Baseline approval was provided by the Kenya Medical Research Institute and the African Medical and Research Foundation (AMREF), Kenya at follow-up. At both phases approval was also granted by the Rift Valley Provincial Medical Officer and the Nakuru district Medical Officer of Health. Approval was sought from the administrative heads in each cluster, usually the village chief. They were also given a copy of the consent form to read and pass on to those in the village.

### Informed consent

Informed consent was obtained from all participants. The objectives of the survey and the examination process were explained to those eligible in the local dialect, in the presence of a witness. A subject was examined only after informed consent was obtained. All participants gave written (or thumbprint) consent to participate.

## Discussion

The Nakuru Eye Disease Cohort Study is the first population-based cohort study of eye disease to have taken place in Africa. It will provide estimates on the incidence of blindness and visual impairment, the incidence and progression of: cataract, refractive error, glaucoma, ARMD and DR as well other retinal conditions. This data will be disseminated to eye care providers and programs in the region to facilitate the provision of eye care services.

## Abbreviations

AMD: Age related macular degeneration; AREDS: Age Related Eye Disease Study; CI: Confidence interval; CSME: Clinically significant macula oedema; D: Dioptres; DR: Diabetic retinopathy; ETDRS: Early Treatment of Diabetic Retinopathy Study; GAT: Goldmann Applanation Tonometry; GPS: Global Positioning System; HbA1c: Haemoglobin 1c; ISGEO: International Society for Geographical Epidemiological Ophthalmology; LogMAR: Logarithm of the minimal angle of resolution; OCO: Ophthalmic clinical officer; PSED: Posterior segment eye diseases; RAAB: Rapid assessment of avoidable blindness; RAPD: Relative Afferent Pupil Defect; RE: Refractive error; SOP: Standard Operating Procedures; VA: Visual acuity; VCDR: Vertical cup to disc ratio; VH: Van-Herrick; VI: Visual impairment; WHO: World Health Organization.

## Competing interests

The authors declare that they have no competing interests.

## Authors’ contributions

Conceptualization and formulation of study protocol; AB, WM, HK, MJB, TP, AF. Training; AB, WM, HR. Monitoring of Study Implementation; AB, HR, MJB, HK. Reading and revising manuscript: MJB, TP, HK, AB, HW, HR, WM, AF. Data cleaning; AB, HW. Data Analysis; AB, HW, HK, MJB. All authors read and approved the final manuscript.

## Pre-publication history

The pre-publication history for this paper can be accessed here:

http://www.biomedcentral.com/1471-2415/14/60/prepub

## Supplementary Material

Additional file 1 **Appendix.** The Nakuru Eye Disease Cohort Study - Study Questionnaire 2013. Click here for file

## References

[B1] Pascolini D Mariotti SP Global estimates of visual impairment: 2010 Br J Ophthalmol 2011 10.1136/bjophthalmol-2011-300539 22133988

[B2] Bastawrous A Hennig BD The global inverse care law: a distorted map of blindness Br J Ophthalmol 2012 10.1136/bjophthalmol-2012-302088 PMC3457914 22740107

[B3] Eloff J Foster A Cataract surgical coverage: results of a population-based survey at Nkhoma, Malawi Ophthalmic Epidemiol 2000 7 3 219 221 10.1076/0928-6586(200009)731-VFT219 11035556

[B4] Kalua K Lindfield R Mtupanyama M Mtumodzi D Msiska V Findings from a rapid assessment of avoidable blindness (RAAB) in Southern Malawi PLoS One 2011 6 4 e19226 10.1371/journal.pone.0019226 21547074 PMC3081843

[B5] Kandeke L Mathenge W Giramahoro C Undendere FP Ruhagaze P Habiyakare C Courtright P Lewallen S Rapid Assessment of Avoidable Blindness in Two Northern Provinces of Burundi without Eye Services Ophthalmic Epidemiol 2012 19 4 211 215 10.3109/09286586.2012.690493 22775276

[B6] Mathenge W Bastawrous A Foster A Kuper H The Nakuru Posterior Segment Eye Disease Study: Methods and Prevalence of Blindness and Visual Impairment in Nakuru, Kenya Ophthalmology 2012 119 10 2033 2039 10.1016/j.ophtha.2012.04.019 22721919

[B7] Mathenge W Kuper H Limburg H Polack S Onyango O Nyaga G Foster A Rapid assessment of avoidable blindness in Nakuru district, Kenya Ophthalmology 2007 114 3 599 605 10.1016/j.ophtha.2006.06.057 17141319

[B8] Mathenge W Nkurikiye J Limburg H Kuper H Rapid assessment of avoidable blindness in Western Rwanda: blindness in a postconflict setting PLoS Med 2007 4 7 e217 10.1371/journal.pmed.0040217 17608561 PMC1904464

[B9] Muller A Zerom M Limburg H Ghebrat Y Meresie G Fessahazion K Beyene K Mathenge W Mebrahtu G Results of a rapid assessment of avoidable blindness (RAAB) in Eritrea Ophthalmic Epidemiol 2011 18 3 103 108 10.3109/09286586.2010.545932 21609238

[B10] Oye JE Kuper H Prevalence and causes of blindness and visual impairment in Limbe urban area, South West Province, Cameroon Br J Ophthalmol 2007 91 11 1435 1439 10.1136/bjo.2007.115840 17389739 PMC2095403

[B11] Rabiu MM Muhammed N Rapid assessment of cataract surgical services in Birnin-Kebbi local government area of Kebbi State, Nigeria Ophthalmic Epidemiol 2008 15 6 359 365 10.1080/09286580802399078 19065428

[B12] Rotchford AP Johnson GJ Rapid assessment of cataract surgical coverage in rural Zululand S Afr Med J = Suid-Afrikaanse tydskrif vir geneeskunde 2000 90 10 1030 1032 11081113

[B13] Oye JE Kuper H Dineen B Befidi-Mengue R Foster A Prevalence and causes of blindness and visual impairment in Muyuka: a rural health district in South West Province, Cameroon Br J Ophthalmol 2006 90 5 538 542 10.1136/bjo.2005.082271 16622082 PMC1857035

[B14] Kikira S RAAB survey of Pemba and Unguja islands, Zanzibar Community Eye Health 2007 20 64 71 18330443 PMC2206324

[B15] Mbulaiteye SM Reeves BC Karabalinde A Ruberantwari A Mulwanyi F Whitworth JA Johnson GJ Evaluation of E-optotypes as a screening test and the prevalence and causes of visual loss in a rural population in SW Uganda Ophthalmic Epidemiol 2002 9 4 251 262 10.1076/opep.9.4.251.1509 12187423

[B16] Kyari F Gudlavalleti MV Sivsubramaniam S Gilbert CE Abdull MM Entekume G Foster A Prevalence of blindness and visual impairment in Nigeria: the National Blindness and Visual Impairment Study Invest Ophthalmol Vis Sci 2009 50 5 2033 2039 10.1167/iovs.08-3133 19117917

[B17] Klein R Klein BE Linton KL De Mets DL The Beaver Dam Eye Study: visual acuity Ophthalmology 1991 98 8 1310 1315 10.1016/S0161-6420(91)32137-7 1923372

[B18] Mitchell P Smith W Attebo K Healey PR Prevalence of open-angle glaucoma in Australia. The Blue Mountains Eye Study Ophthalmology 1996 103 10 1661 1669 10.1016/S0161-6420(96)30449-1 8874440

[B19] Vingerling JR Dielemans I Hofman A Grobbee DE Hijmering M Kramer CF De Jong PT The prevalence of age-related maculopathy in the Rotterdam Study Ophthalmology 1995 102 2 205 210 10.1016/S0161-6420(95)31034-2 7862408

[B20] Buch H Vinding T La Cour M Nielsen NV The prevalence and causes of bilateral and unilateral blindness in an elderly urban Danish population. The Copenhagen City Eye Study Acta Ophthalmol Scand 2001 79 5 441 449 10.1034/j.1600-0420.2001.790503.x 11594976

[B21] Leske MC Connell AM Schachat AP Hyman L The Barbados Eye Study Prevalence of open angle glaucoma Arch Ophthalmol 1994 112 6 821 829 10.1001/archopht.1994.01090180121046 8002842

[B22] Delcourt C Diaz JL Ponton-Sanchez A Papoz L Smoking and age-related macular degeneration. The POLA Study. Pathologies Oculaires Liees a l'Age Arch Ophthalmol 1998 116 8 1031 1035 10.1001/archopht.116.8.1031 9715683

[B23] Livingston PM Carson CA Stanislavsky YL Lee SE Guest CS Taylor HR Methods for a population-based study of eye disease: the Melbourne Visual Impairment Project Ophthalmic Epidemiol 1994 1 3 139 148 10.3109/09286589409047222 8790620

[B24] Miyazaki M Kubota T Kubo M Kiyohara Y Iida M Nose Y Ishibashi T The prevalence of pseudoexfoliation syndrome in a Japanese population: the Hisayama study J Glaucoma 2005 14 6 482 484 10.1097/01.ijg.0000185436.15675.b3 16276281

[B25] Sasaki H Jonasson F Kojima M Katoh N Ono M Takahashi N Sasaki K The Reykjavik Eye Study–prevalence of lens opacification with reference to identical Japanese studies Ophthalmologica 2000 214 6 412 420 10.1159/000027535 11054002

[B26] Azen SP Varma R Preston-Martin S Ying-Lai M Globe D Hahn S Binocular visual acuity summation and inhibition in an ocular epidemiological study: the Los Angeles Latino Eye Study Invest Ophthalmol Vis Sci 2002 43 6 1742 1748 12036974

[B27] Mathenge W Bastawrous A Peto T Leung I Foster A Kuper H Prevalence of age-related macular degeneration in nakuru, kenya: a cross-sectional population-based study PLoS Med 2013 10 2 e1001393 10.1371/journal.pmed.1001393 23431274 PMC3576379

[B28] Bastawrous A Mathenge W Foster A Kuper H Prevalence and predictors of refractive error and spectacle coverage in Nakuru, Kenya: a cross-sectional, population-based study Int Ophthalmol 2013 10.1007/s10792-013-9742-6 23440405

[B29] Sherwin JC Keeffe JE Kuper H Islam FM Muller A Mathenge W Functional presbyopia in a rural Kenyan population: the unmet presbyopic need Clin Experiment Ophthalmol 2008 36 3 245 251 10.1111/j.1442-9071.2008.01711.x 18412593

[B30] Mathenge W Foster A Kuper H Urbanization, ethnicity and cardiovascular risk in a population in transition in Nakuru, Kenya: a population-based survey BMC Public Health 2010 10 569 10.1186/1471-2458-10-569 20860807 PMC2956724

[B31] Ploubidis GB Mathenge W De Stavola B Grundy E Foster A Kuper H Socioeconomic position and later life prevalence of hypertension, diabetes and visual impairment in Nakuru, Kenya Int J Public Health 2013 58 1 133 141 10.1007/s00038-012-0389-2 22814479

[B32] Statistics KKNBo Kenya 2009 Population and Housing census highlights 2010

[B33] Bureau USC Demographic Data for Kenya: International Data Base IDB Sum 2005

[B34] Turner AG Magnani RJ Shuaib M A not quite as quick but much cleaner alternative to the Expanded Programme on Immunization (EPI) Cluster Survey design Int J Epidemiol 1996 25 1 198 203 10.1093/ije/25.1.198 8666490

[B35] Kuper H Polack S Eusebio C Mathenge W Wadud Z Foster A A case–control study to assess the relationship between poverty and visual impairment from cataract in Kenya, the Philippines, and Bangladesh PLoS Med 2008 5 12 e244 10.1371/journal.pmed.0050244 19090614 PMC2602716

[B36] Bourne RR Rosser DA Sukudom P Dineen B Laidlaw DA Johnson GJ Murdoch IE Evaluating a new logMAR chart designed to improve visual acuity assessment in population-based surveys Eye (Lond) 2003 17 6 754 758 10.1038/sj.eye.6700500 12928690

[B37] Rosser DA Laidlaw DA Murdoch IE The development of a "reduced logMAR" visual acuity chart for use in routine clinical practice Br J Ophthalmol 2001 85 4 432 436 10.1136/bjo.85.4.432 11264133 PMC1723918

[B38] Bourne RR Dineen B Modasser Ali S Mohammed Noorul Huq D Johnson GJ The National Blindness and Low Vision Prevalence Survey of Bangladesh: research design, eye examination methodology and results of the pilot study Ophthalmic Epidemiol 2002 9 2 119 132 10.1076/opep.9.2.119.1520 11821977

[B39] Bourne R Dineen B Jadoon Z Lee PS Khan A Johnson GJ Foster A Khan D The Pakistan national blindness and visual impairment survey–research design, eye examination methodology and results of the pilot study Ophthalmic Epidemiol 2005 12 5 321 333 10.1080/09286580500230948 16272052

[B40] Van Herick W Shaffer RN Schwartz A Estimation of width of angle of anterior chamber. Incidence and significance of the narrow angle Am J Ophthalmol 1969 68 4 626 629 5344324 10.1016/0002-9394(69)91241-0

[B41] Shaffer RN Schwartz A Gonioscopy Surv Ophthalmol 1957 2 5 389 409 13486432

[B42] Narayanaswamy A Sakata LM He MG Friedman DS Chan YH Lavanya R Baskaran M Foster PJ Aung T Diagnostic performance of anterior chamber angle measurements for detecting eyes with narrow angles: an anterior segment OCT study Arch Ophthalmol 2010 128 10 1321 1327 10.1001/archophthalmol.2010.231 20938002

[B43] Thylefors B Chylack LT Jr Konyama K Sasaki K Sperduto R Taylor HR West S A simplified cataract grading system Ophthalmic Epidemiol 2002 9 2 83 95 10.1076/opep.9.2.83.1523 11821974

[B44] NHS S Diabetic Retinopathy Screening Services in Scotland: Recommendations for Implementation 2003

[B45] World_Health_Organization Coding instructions for the WHO/PBL eye examination record (version III) 1988 vol: PBL

[B46] Foster PJ Buhrmann R Quigley HA Johnson GJ The definition and classification of glaucoma in prevalence surveys Br J Ophthalmol 2002 86 2 238 242 10.1136/bjo.86.2.238 11815354 PMC1771026

[B47] Wang YX Hu LN Yang H Jonas JB Xu L Frequency and associated factors of structural progression of open-angle glaucoma in the Beijing Eye Study Br J Ophthalmol 2012 96 6 811 815 10.1136/bjophthalmol-2011-301224 22408234

[B48] Davis MD Gangnon RE Lee LY Hubbard LD Klein BE Klein R Ferris FL Bressler SB Milton RC The Age-Related Eye Disease Study severity scale for age-related macular degeneration: AREDS Report No. 17 Arch Ophthalmol 2005 123 11 1484 1498 16286610 10.1001/archopht.123.11.1484 PMC1472813

[B49] Grading diabetic retinopathy from stereoscopic color fundus photographs--an extension of the modified Airlie House classification. ETDRS report number 10. Early Treatment Diabetic Retinopathy Study Research Group Ophthalmology 1991 98 5 786 806 2062513

