# Supplementary material for: The Nakuru eye disease cohort study: methodology & rationale
Source: BMC Ophthalmol. 2014 May 1;14:60. doi: 10.1186/1471-2415-14-60 (PMC4024270; doi:10.1186/1471-2415-14-60)
Supplement: Additional file 1 — Appendix. The Nakuru Eye Disease Cohort Study - Study Questionnaire 2013. [file 1471-2415-14-60-S1.pdf]

Study ID ☐☐☐-☐☐

The Nakuru Eye Disease Cohort Study

-

Study Questionnaire

2013

REFERRALS		
DIABETIC	☐ Yes – **REFER**	**READING GLASSES DISPENSED? POWER**
Distance GLASSES	☐ Yes - **REFER**	
CATARACT	☐ Yes - **REFER**	**Any other treatment? E.g. drops**
GLAUCOMA	☐ Yes - **REFER**	
DIABETIC RETINOPATHY	☐ Yes - **REFER**	
OTHER	☐ Yes - **REFER**	

Study ID ☐☐☐-☐☐

Mark Tick Boxes using a black biro with a cross “X”, if marked incorrectly, fill in the box and mark the correct box.

e.g. ☒ if incorrect fill the box → ■

Answer Questions in the grey boxes

Phase	Section	Page	Complete?
1	Registration/Demographic Data [A]	3	☐
	Autorefraction [B]	4	☐

Phase	Visual Acuity [A]	Interview [B]	Height/Weight etc. [C]	Slit Lamp – Undilated [D]	Visual Fields [E]
2	☐	☐	☐	☐	☐
Page	5-7	8-12	13	14-16	17

All tests above this line must be completed before pupil dilation

Phase	Section	Page	Complete?
3	Dilated Slit Lamp Examination	18-22	☐
	Fundus Camera	23	☐

Study ID ☐☐☐-☐☐

1. A. Demographic data (Registration Desk)

**Cluster Number**	3 digit number 001-100	
**Individual Number**	2 digit number 01-50	
**Study ID Number**	Cluster Number – Individual Number	☐☐☐ -- ☐☐
**Date of the examination**	Day / Month / Year (dd/mm/yyyy)	
**First name**	text	
**Last name**	text	
**Common name**	text	
**ID Number**	from ID Card if available	
**Serial Number**	from ID Card if available	
**Village name**		
**Telephone number**		
**Whose telephone is this?**		
**Sex**	☐ Male (1)	☐ Female (2)
**Date of birth**	Date / Month / Year<br>If year only known enter 99/99/19YY	dd | mm | 19 | yy
**Age**	in years (55+)	
**Ask "mother tongue"**	☐ Kikuyu (1)	☐ Kalenjin (2)
	☐ Kisii (3)	☐ Luo (4)
	☐ Luhyia (5)	☐ Masaai (6)
	☐ Kamba (7)	☐ Other (8)

**Highest level of Education**	☐ Primary (1)	☐ Secondary (2)	☐ College/University (3)	☐ None (4)

I have recorded the data onto the form:

Name **Date**

Study ID ☐☐☐-☐☐

1. B. Refraction (Ophthalmic Nurse)

Refraction?<br><br>(Select ONE only)	**Right Eye**	**Left Eye**
	☐ AutoRefraction possible	☐ AutoRefraction possible
	☐ AutoRefraction not possible	☐ AutoRefraction not possible
	☐ Manual Refraction	☐ Manual Refraction
Refraction Result	**Right Eye**	**Left Eye**
Sphere +/- 00.00 (to nearest 0.25)		
Cylinder +/- 00.00 (to nearest 0.25)		
Axis 0-180		
Reliability score (1 to 9)		

Print out Autorefraction and attach to back of booklet

Study ID ☐☐☐-☐☐

2.A. Presenting Vision (Ophthalmic Nurse)

Glasses<br>(Select **ONE** only)	☐ Wearing distance glasses (Go to 2.A.a)
	☐ Has no distance glasses (Go to 2.A.b)
	☐ Forgot distance glasses (Go to 2.A.b)

Wears glasses for reading	☐ Yes	☐ No
Wears aphakic glasses (has had cataract surgery)	☐ Yes	☐ No

2.A.a

Test vision WITH glasses if available. If own glasses not available skip to Question 2.A.b (page 6).

		R	L
Vision WITH distance or aphakic glasses at 4m	Number of letters seen at 4 meters (0 to 39)		
If Visual Acuity Recorded at 4 meters (greater than 00) in either eye move to next station<br>*If misses top E at 4m, move to 1m and retest (Record 00 at 4m)*			

		R	L
Vision WITH distance or aphakic glasses at 1m	Number of letters seen at 1 meter (0 to 39)		
If misses top E at 1m, move to next box (Record 00 at 1m)			

If cannot see at 1m<br><br>*(Select ONE only)*	**Right Eye**	**Left Eye**
	☐ Counting fingers at 1m	☐ Counting fingers at 1m
	☐ Hand Movements	☐ Hand Movements
	☐ Perception of light	☐ Perception of light
	☐ No light perception (in dark)	☐ No light perception (in dark)

Study ID ☐☐☐-☐☐

2.A.b (not needed if vision was tested with own glasses)

		R	L
Vision WITHOUT glasses at 4m	Number of letters seen at 4 meters (0 to 39)		
If Visual Acuity Recorded at 4 meters (greater than 00) in either eye move to next station<br>*If misses top E at 4m, move to 1m and retest (Record 00 at 4m)*			

		R	L
Vision WITHOUT glasses at 1m (if 00 at 4m)	Number of letters seen at 1 meter (0 to 39)		
If misses top E at 1m, move to next box (Record 00 at 1m)			

If cannot see at 1m<br><br>*(Select ONE only)*	**Right Eye**	**Left Eye**
	☐ Counting fingers at 1m	☐ Counting fingers at 1m
	☐ Hand Movements	☐ Hand Movements
	☐ Perception of light	☐ Perception of light
	☐ No light perception (in dark)	☐ No light perception (in dark)

Study ID ☐☐☐-☐☐

2.A.c

Is Best Corrected Visual Acuity (Wearing refraction results) Indicated From Page 4 (Select **ONE** only)	**Vision in Best Eye**
	☐ Not indicated (could read 25 or more letters in the best eye) ***Move patient to next station*** *(miss page 7)*
	☐ Indicated (could not read 25 or more letters in the best eye, **refraction not available therefore use pinhole.**

NOW TEST BEST CORRECTED/PIN HOLE VISUAL ACUITY IF LESS THAN 25 LETTERS SEEN IN BETTER EYE

How was corrected vision tested (Select **ONE** only)	☐CORRECTED WITH LENSES
	☐CORRECTED WITH PINHOLE<br>If refraction was not possible
	☐CORRECTED VISION NOT TESTED

Using refraction result from Section 1.B, page 4.

BEST CORRECTED VISUAL ACUITY at 4m	Number of letters seen at 4 meters (0 to 39)	**R**	**L**
Move to next station if Best Corrected Visual Acuity Recorded at 4 meters (greater than 00)<br>*If misses top E at 4m, move to 1m and retest (Record 00 at 4m)*			

BEST CORRECTED VISUAL ACUITY at 1m	Number of letters seen at 1 meter (0 to 39)	**R**	**L**
If misses top E at 1m, Record 00 at 1m			

I have recorded the data onto the form:

Name **Date**

Study ID ☐☐☐-☐☐

2.B. General Heath (Nurse/Interviewer)

Have you ever been diagnosed with diabetes?	☐ Yes (1)		☐ No (2)		
					If NO, go to next question
How long ago were you diagnosed with diabetes?	Years (01 – 99)<br>If less than one year, enter "01"				
Are you receiving treatment for diabetes? (select **ALL** that apply)	☐ Yes, insulin (1)	☐ Yes, tablets (2)	☐ Yes, diet (3)	☐ Yes, traditional (4)	☐ No (5)

Have you ever been diagnosed with high blood pressure?	☐ Yes (1)		☐ No (2)	
				If NO, go to next question
How long ago were you diagnosed with high blood pressure?	Years (if less than 1 year mark "0"			
Are you receiving treatment for high blood pressure? (select **ALL** that apply)	☐ Yes, tablets (1)	☐ Yes, diet (2)	☐ Yes, traditional (3)	☐ No (4)

Have you been diagnosed or are you suffering from any of the following? (tick all that apply)	☐ Renal Disease (1)	☐ Heart Disease (2)	☐ Foot Ulcers (3)	☐ None (4)
Did/Do your **mother** have any of the following?	☐ Diabetes (1)	☐ High Blood Pressure (2)	☐ Blinding eye condition (3)	☐ Not sure/None (4)
Did/Do your **father** have any of the following?	☐ Diabetes (1)	☐ High Blood Pressure (2)	☐ Blinding eye condition (3)	☐ Not sure/None (4)
Did/Do your **sibling**s have any of the following?	☐ Diabetes (1)	☐ High Blood Pressure (2)	☐ Blinding eye condition (3)	☐ Not sure/None (4)

2.B. continued: Blood Pressure (Nurse)

Take First and Second Blood Pressure Measurements			
First Blood Pressure Reading	Systolic (00 to 250)	Diastolic (00 to 250)	Pulse (00 to 250)
Wait ten minutes between readings. Ensure patient is resting, sitting and no talking whilst BP being taken			
Second Blood Pressure Reading	Systolic (00 to 250)	Diastolic (00 to 250)	Pulse (00 to 250)

Do you drink alcohol? (Answer **ONE** only)	☐ Daily/Most days (1)	☐ Weekends only (2)	☐ 1-2 times per month (3)	☐ Special occasions only (4)	☐ Never (5)	☐ Former (>6 months) (6)

Have you ever smoked?	☐ Never (1)	☐ Former (stopped > 6 months ago) (2)	☐ Current (in last 6 months) (3)
If "Never", skip to next question			
	Age at starting	years	
	Duration of use	years	
	Number of days per week	days (max 07)	
	Number smoked per day		

Have you ever snuffed tobacco?	☐ Never (1)	☐ Former (stopped > 6 months ago) (2)	☐ Current (in last 6 months) (3)
If "Never", skip to next question			
	Age at starting	years	
	Duration of use	years	
	How many days used per week	days (max 07)	
	How many times used per day		

2.B. continued

Have you ever chewed tobacco?	☐ Never (1)	☐ Former (stopped > 6 months ago) (2)	☐ Current (in last 6 months) (3)
If "Never", skip to next page			
	Age at starting	years	
	Duration of use	years	
	Number of days per week	days (max 07)	
	Number chewed per day		

Study ID ☐☐☐-☐☐

2.B. Socioeconomic Status (Nurse/Interviewer)

In the last month have you had a job other than working in the field owned or rented by the household?	☐ Yes (1)	☐ No (2)

Major external wall material of your home<br>(Select **ONE** only)	☐ Brick (1)	☐ Concrete Block (2)	☐ Stone (3)
	☐ Unbaked brick (4)	☐ Wood/logs (5)	☐ Tin, zinc sheeting (6)
	☐ Flattened tin cans (7)	☐ Mud (8)	☐ Stone and Mud (9)
	☐ Canvas/Felt (10)	☐ Other (11)	
Primary Roof Material of your home<br>(Select **ONE** only)	☐ Concrete (1)	☐ Shingles (2)	☐ Asbestos Sheets (3)
	☐ Metal Sheets (4)	☐ Tile (5)	☐ Wood (6)
	☐ Unbaked bricks (7)	☐ Thatch (8)	☐ Other (9)
Primary Floor Material of your home<br>(Select **ONE** only)	☐ Parquet (1)	☐ Painted wood (2)	☐ Tile (3)
	☐ Linoleum (4)	☐ Concrete (5)	☐ Clay/earthen floor (6)
	☐ Other (7)		

Where is the toilet?<br>(Select **ONE** only)<br>If more than one toilet mark best one	☐ Inside dwelling (1)	☐ Outside dwelling – in compound (2)
	☐ Outside dwelling – outside compound (3)	☐ Not Applicable/no access to a toilet (uses bush etc) (8)

Type of toilet?<br>(Select **ONE** only)<br>If more than one toilet mark best one	☐ Flush Toilet (1)	☐ Traditional latrine (2)	☐ Improved pit latrine with ventilation (VIP)(3)
	☐ Bowl/Bucket (4)	☐ Other (5)	☐ No toilet (6)

Study ID ☐☐☐-☐☐

Household assets<br>(Select **ALL** that apply)	☐ Radio/Hifi	☐ Sewing machine
	☐ TV/VCR/DVD	☐ Table
	☐ Fridge/Freezer	☐ Bicycle
	☐ Telephone/cell phone	☐ Washing machine
	☐ Cupboard	☐ Motor vehicle/car
	☐ Sofaset/armchair	☐ Motorbike
How many of the following animals do you possess?<br>*Enter "0" if none*	Cows?	
	Sheep?	
	Pigs?	
	Chicken/ducks?	

Blood Pressure

	Systolic (00 to 250)	Diastolic (00 to 250)	Pulse (00 to 250)
Third Blood Pressure Reading			

Treatment history and barriers to uptake (Nurse/Interviewer)

Previous Eye Surgery<br>(Select **ALL** that apply)	**Right Eye**	**Left Eye**
	☐ Cataract Surgery	☐ Cataract Surgery
	☐ Eye lid surgery (Trachoma)	☐ Eye lid surgery (Trachoma)
	☐ Glaucoma Surgery	☐ Glaucoma Surgery
	☐ Other	☐ Other
	☐ No Surgery	☐ No Surgery

Current regular medicine for the eyes<br>(Select **ALL** that apply)	**Right or Left Eyes**
	☐ Antibiotics
	☐ Steroids
	☐ Anti-Glaucoma
	☐ Lubricant
	☐ Other
	☐ No medicines

Study ID ☐☐☐-☐☐

2.C. Anthropometry (Nurse)

**Patient Age from page 3:**	

Height in cm (no dp)		888: unable, 999: refused	
Weight in kg (1 dp)		888: unable, 999: refused	
Body Fat %		888: unable, 999: refused	
Muscle Mass		888: unable, 999: refused	
Bone MASS		888: unable, 999: refused	
Metabolic Age		888: unable, 999: refused	
Total Body Water %		888: unable, 999: refused	
Visceral Fat Level		01-59<br>888: unable, 999: refused	
Waist circumference **in cm** (no dp)		888: unable, 999: refused	
Hip circumference **in cm** (no dp)		888: unable, 999: refused	

Random blood sugar mmol/L	0.0 to 35.0	

If ≥ 11.1 mmol/l	HbA1c (%)	Enter number on screen (<4 or >13 may be shown)	

I have recorded the data onto the form:

Name **Date**

Study ID ☐☐☐-☐☐

2.D. Anterior Segment Examination (Ophthalmologist)

Relative Afferent Pupil Defect	**Right Eye**		**Left Eye**	
	☐ Definite	☐ Subtle	☐ Definite	☐ Subtle
	☐ No	☐ Not able	☐ No	☐ Not able

Pterygium present and extent	**Right Eye**	**Left Eye**
	☐ No Pterygium	☐ No Pterygium
	☐ Pterygium – Cornea NOT involved	☐ Pterygium – Cornea NOT involved
	☐ Pterygium – Cornea Involved	☐ Pterygium – Cornea Involved
mm in to cornea from limbus (0-12)	☐☐	☐☐

Study ID ☐☐☐-☐☐

2.D. continued

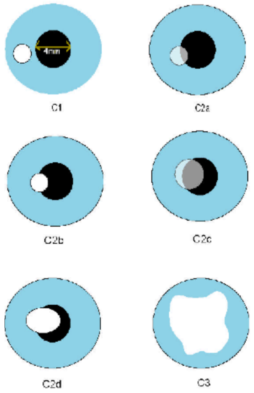

Corneal scarring grade	Right Eye	Left Eye
	☐ NO Opacity	☐ NO Opacity
	☐ Opacity not entering central 4mm (C1)	☐ Opacity not entering central 4mm (C1)
	☐ Opacity within central 4mm but not entering within the central 1mm of the cornea. The pupil margin is visible through the opacity (C2a)	☐ Opacity within central 4mm but not entering within the central 1mm of the cornea. The pupil margin is visible through the opacity (C2a)
	☐ Opacity within central 4mm but not entering within the central 1mm of the cornea. The pupil margin is not visible through the opacity (C2b)	☐ Opacity within central 4mm but not entering within the central 1mm of the cornea. The pupil margin is not visible through the opacity (C2b)
	☐ Opacity within central 4mm and entering the central 1mm of the cornea. The pupil margin is visible through the opacity (C2c)	☐ Opacity within central 4mm and entering the central 1mm of the cornea. The pupil margin is visible through the opacity (C2c)
	☐ Opacity within central 4mm and entering within the central 1mm of the cornea. The pupil margin is not visible through the opacity (C2d)	☐ Opacity within central 4mm and entering within the central 1mm of the cornea. The pupil margin is not visible through the opacity (C2d)
	☐ Opacity large enough and dense enough to make whole pupil margin invisible (C3)	☐ Opacity large enough and dense enough to make whole pupil margin invisible (C3)
	☐ Phthisis (C4)	☐ Phthisis (C4)

Anterior Segment	Right Eye	Left Eye
Mark **ALL** that apply	☐ Pseudoexfoliation	☐ Pseudoexfoliation
	☐ Iris Trans illumination	☐ Iris Trans illumination
	☐ Krukenberg's Spindle	☐ Krukenberg's Spindle
	☐ Evidence of previous inflammation	☐ Evidence of previous inflammation
	☐ None of the above	☐ None of the above

Study ID ☐☐☐-☐☐

2.D. continued

Van Herick's	Right Eye	Left Eye
	☐ ACD = 0 or negligible (0)	☐ ACD = 0 or negligible (0)
	☐ ACD ≤1/4 cornea (1)	☐ ACD ≤1/4 cornea (1)
	☐ ACD = 1/4 cornea (2)	☐ ACD = 1/4 cornea (2)
	☐ ACD =1/4-1/2 cornea (3)	☐ ACD =1/4-1/2 cornea (3)
	☐ ACD >=1/2 cornea (4)	☐ ACD >=1/2 cornea (4)
	☐ not gradable (9)	☐ not gradable (9)

Applanation IOP (mmHg)	99 = not possible	**R**	**L**

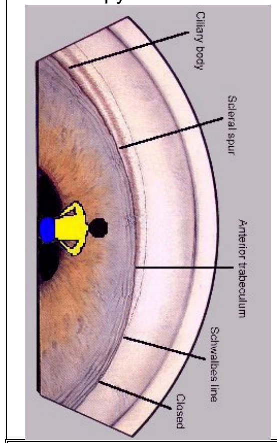

Gonioscopy	Right Eye	Left Eye
	☐ Nil (0)	☐ Nil (0)
	☐ Scwalbe's line and anterior meshwork (1)	☐ Scwalbe's line and anterior meshwork (1)
	☐ Posterior pigmented meshwork (2)	☐ Posterior pigmented meshwork (2)
	☐ Scleral Spur (3)	☐ Scleral Spur (3)
	☐ Ciliary Band (4)	☐ Ciliary Band (4)
	☐ Not gradable (5)	☐ Not gradable (5)

Safe to dilate? ☐ Yes ☐ No

Study ID ☐☐☐-☐☐

2.E. Visual Fields (Visual Field Technician)

VF completed?	Right Eye	Left Eye
	☐ Yes	☐ Yes
	☐ No – uncooperative	☐ No – uncooperative
	☐ No – poor visual acuity	☐ No – poor visual acuity
	☐ No – machine failure	☐ No – machine failure
Classification (in the field – by ophthalmologist or OCO)	**Right Eye**	**Left Eye**
	☐ Normal	☐ Normal
	☐ Abnormal – definite Glaucoma	☐ Abnormal – definite Glaucoma
	☐ Abnormal – suspect glaucoma	☐ Abnormal – suspect glaucoma
	☐ Abnormal – non-glaucoma	☐ Abnormal – non-glaucoma

Print out Visual Fields and attach to back of booklet

I have recorded the data onto the form and printed the visual fields:

Name **Date**

Study ID ☐☐☐-☐☐

3.A. Dilated Examination (Ophthalmologist)

WHO CATARACT GRADING (See Document for Reference)

Cataract<br>(Select ONE only)<br>3.A.1	Right Eye	Left Eye
	[0] ☐ No cataract	[0] ☐ No cataract
	[1] ☐ Gradable cataract	[1] ☐ Gradable cataract
	[2] ☐ Mature	[2] ☐ Mature
	[3] ☐ Hypermature	[3] ☐ Hypermature
	[4] ☐ Corneal Opacity	[4] ☐ Corneal Opacity
	[5] ☐ Phythsis	[5] ☐ Phythsis
	[7] ☐ Aphakia	[7] ☐ Aphakia
	[8] ☐ IOL	[8] ☐ IOL
	[9] ☐ Can not grade	[9] ☐ Can not grade
Nuclear<br>(Select ONE only)	**Right Eye**	**Left Eye**
	☐ Not applicable	☐ Not applicable
	☐ Nuclear 0 [0]	☐ Nuclear 0 [0]
	☐ Nuclear 1 [1]	☐ Nuclear 1 [1]
	☐ Nuclear 2 [2]	☐ Nuclear 2 [2]
	☐ Nuclear 3 [3]	☐ Nuclear 3 [3]
	☐ Aphakia [7]	☐ Aphakia [7]
	☐ IOL [8]	☐ IOL [8]
	☐ Cannot grade [9]	☐ Cannot grade [9]

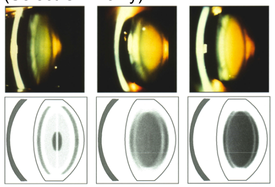

1 2 3

Study ID ☐☐☐-☐☐

3.A. continued

Cortical<br>(Select ONE only)<br>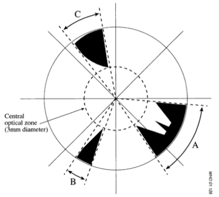<br>0: <1/8, 1: 1/8 to <1/4, 2: ¼ to <1/2, 3: 1/2+	**Right Eye**			**Left Eye**		
	☐ Not applicable			☐ Not applicable		
	☐ Cortical 0 [0]			☐ Cortical 0		
	☐ Cortical 1 [1]			☐ Cortical 1		
	☐ Cortical 2 [2]			☐ Cortical 2		
	☐ Cortical 3 [3]			☐ Cortical 3		
	☐ Aphakia [7]			☐ Aphakia		
	☐ IOL [8]			☐ IOL		
	☐ Cannot grade [9]			☐ Cannot grade		
Cortical Central? (central 3mm)	☐ Yes [1]	☐ No [2]	☐ N/A [3]	☐ Yes [1]	☐ No [2]	☐ N/A [3]

Posterior Subcapsular (PSC)	**Right Eye**	**Left Eye**
(Select ONE only)	☐ Not applicable	☐ Not applicable
0: <1mm	☐ PSC 0 [0]	☐ PSC 0 [0]
1: >=1mm, <2mm	☐ PSC 1 [1]	☐ PSC 1 [1]
2: >=2mm, <3mm	☐ PSC 2 [2]	☐ PSC 2 [2]
3: >=3mm	☐ PSC 3 [3]	☐ PSC 3 [3]
	☐ Aphakia [7]	☐ Aphakia [7]
	☐ IOL [8]	☐ IOL [8]
	☐ Cannot grade [9]	☐ Cannot grade [9]
Posterior Capsular Opacification (PCO) with IOL	**Right Eye**	**Left Eye**
	☐ Yes - within central 3mm [1]	☐ Yes - within central 3mm [1]
	☐ No – Clear capsule [2]	☐ No – Clear capsule [2]
	☐ Not sure [3]	☐ Not sure [3]
	☐ Evidence of capsulotomy [4]	☐ Evidence of capsulotomy [4]
	☐ Yes – outside central 3mm [5]	☐ Yes – outside central 3mm [5]
	☐ N/A [9]	☐ N/A [9]

Study ID ☐☐☐-☐☐

3.A. continued

**POSTERIOR SEGMENT EXAMINATION (1 in 10 participants and those in whom imaging not possible)**		
☐ 1 in 10	☐ Imaging not possible	☐ Not applicable *(skip to 3B)*

View of PSED at slit lamp	**Right Eye**	**Left Eye**
	☐ Clear	☐ Clear
	☐ Hazy	☐ Hazy
	☐ No view	☐ No view

Vertical Cup to Disc Ratio	0.0 to 1.0	R	☐Can not assess	L	☐Can not assess

VCDR asymmetry (>=0.2)	**Both Eyes**	
	☐ Yes [1]	
	☐ No [2]	
	☐ Can not assess [3]	
Disc Haemorrhage	**Right Eye**	**Left Eye**
	☐ Yes [1]	☐ Yes [1]
	☐ No [2]	☐ No [2]
	☐ Can not assess [3]	☐ Can not assess [3]
Disc Notch	**Right Eye**	**Left Eye**
	☐ Yes [1]	☐ Yes [1]
	☐ No [2]	☐ No [2]
	☐ Can not assess [3]	☐ Can not assess [3]
Disc Atrophy	**Right Eye**	**Left Eye**
	☐ Yes [1]	☐ Yes [1]
	☐ No [2]	☐ No [2]
	☐ Can not assess [3]	☐ Can not assess [3]

Study ID ☐☐☐-☐☐

3.A. continued

Diabetic Retinopathy (Select **ONE** only)	**Right Eye**	**Left Eye**
	☐ No diabetic retinopathy	☐ No diabetic retinopathy
	☐ Non-proliferative	☐ Non-proliferative
	☐ Proliferative/end stage	☐ Proliferative/end stage
	☐ Cannot assess	☐ Cannot assess
Diabetic Maculopathy (Select **ONE** only)	☐ Diabetic Maculopathy	☐ Diabetic Maculopathy
	☐ No Diabetic Maculopathy	☐ No Diabetic Maculopathy
	☐ Cannot assess	☐ Cannot assess
Age Related Maculopathy (ARM)	**Right Eye**	**Left Eye**
	☐ No ARM [1]	☐ No ARM [1]
	☐ Drusen [2]	☐ Drusen [2]
	☐ Hypo/hyper pigmentation [3]	☐ Hypo/hyper pigmentation [3]
	☐ Can not assess [4]	☐ Can not assess [4]
Age Related Macular Degeneration (ARMD)	**Right Eye**	**Left Eye**
	☐ No ARMD	☐ No ARMD
	☐ Dry or Geographic	☐ Dry or Geographic
	☐ Wet/Neovascular/Disciform	☐ Wet/Neovascular/Disciform
	☐ Can not assess	☐ Can not assess
Other PSED Pathology	**Right Eye**	**Left Eye**
	☐ Yes	☐ Yes
	☐ No	☐ No
	☐ Can not assess	☐ Can not assess
If Yes - Specify (free text)	**R**	**L**

Study ID ☐☐☐-☐☐

3.B. Fundus Photography

**Participant details entered on home screen**	☐ Yes	☐ No
**Participant Study ID Number**	**XXX-XX**	☐☐☐-☐☐
Camera Failure?	☐ Yes	☐ No

**ANTERIOR SEGMENT & LENS PHOTO**<br>*SIT PATIENT BACK SLIGHTLY ON CHIN REST (3cm)*		
Image clarity	**Right Eye**	**Left Eye**
	☐ Clear	☐ Clear
	☐ Hazy	☐ Hazy
	☐ No view	☐ No view

**FUNDUS PHOTOGRAPH**<br>*AUTOMATIC MODE*<br>*MANUAL IF UNABLE*		
Posterior Segment Image clarity	**Right Eye**	**Left Eye**
	☐ Clear	☐ Clear
	☐ Hazy	☐ Hazy
	☐ No view	☐ No view

<u>Participant information sheet – to be translated in to Kiswahili and read to participants</u>

THE INCIDENCE AND PROGRESSION OF POSTERIOR SEGMENT EYE DISEASE IN NAKURU COUNTY IN RESIDENTS AGED 55 YEARS AND ABOVE

You are being invited to take part in a research study. Before you decide to take part, it is important for you to understand why the research is being done and what it will involve. I will read information to you about this study. Please ask me if there is anything that is not clear or if you would like more information. Take time to decide whether or not you wish to take part

What is the purpose of the study?

In the world today there are about 39 million blind people. Approximately half of these people are blind due to cataract, making this the single largest cause of global blindness. In 2007/8 we undertook a survey to investigate how common eye diseases are among older people in Nakuru. This information helps us to plan health services more efficiently. We now want to follow-up the people we examined to see how quickly eye disease progresses and how often new eye disease occurs.

Why have I been chosen?

Every person who was randomly selected for the study 5 years ago (in 2007/2008) is being invited to take part again in the study so we can see what has happened to you over this time,

Do I have to take part?

No. It is up to you to decide whether or not to take part. If you decide to take part you will be asked to sign a consent form. If you decide to take part you are still free to withdraw at any time and without giving a reason.

What is involved in the study?

We will give you a very complete eye examination to look for any problem at the front and inside the eye using several machines. We will also check the pressure in your eye. Some of the examinations will involve contact with your eye. Some drops will be put in your eye so that you feel no pain. We will also put drops in your eyes to make the pupils as big as possible so we can see inside clearly. This will cause blurring of your near vision for a few hours afterwards and so you will not be able to drive or operate dangerous machinery for the rest of the day. The risks and likelihood of side-effects from this procedure is extremely small. We will also collect information about your history of diabetes, high blood pressure, eye diseases, smoking and alcohol all of which affect your eyes.

Your height, weight and blood pressure will be measured. A finger prick blood sample will be taken to check for diabetes, and a swab from the inside of your cheek will be taken to measure genetic material (DNA). This genetic material carries information for making up our bodies and is different in all people. Having DNA samples helps us to understand whether diseases run in the family. The results are unlikely to have any implications for you personally. We will store the DNA for future laboratory research that may be needed.

All information which is collected about you during the course of the research will be kept strictly confidential and your name will never be released.

Should we find that you could benefit from ant further eye treatment we will arrange an appointment for you to have treatment done at Nakuru eye unit. If you are found to be diabetic or have high blood pressure we will arrange for you be seen at the Nakuru provincial hospital. You will have to pay normal hospital fees for some of the treatment at the hospital. It is up to you to decide whether you would like to take up the offer of treatment or not.

CONSENT FORM

The information sheet concerning this study has been read to me, and I understand what will be expected of me if I take part in this study.

My questions concerning this study have been answered by ______________________. I understand that participation in this study is voluntary. I also understand that I may withdraw from this study at any time without giving a reason and that this will not affect my normal care.

I agree to take part in this study.

I agree to have a sample of genetic material (DNA) taken: Yes/No

Name of study subject: ______________________________

Signature or thumbprint ______________________________

Witness ______________________________

Date ______________________________

* <u>Witness</u>: By signing in this column I warrant that I have read this form and the information form to the persons against whose names my signature appears. I am sure that each of these persons has understood what is required of him/her and has agreed to take part in the study.
